# Identification of *microRNA158* from *Anthurium andraeanum* and Its Function in Cold Stress Tolerance

**DOI:** 10.3390/plants11233371

**Published:** 2022-12-04

**Authors:** Li Jiang, Yanxia Fu, Pan Sun, Xingkai Tian, Guangdong Wang

**Affiliations:** Key Laboratory of Landscaping, Ministry of Agriculture and Rural Affairs, Key Laboratory of Biology of Ornamental Plants in East China, National Forestry and Grassland Administration, Department of Horticulture, Nanjing Agricultural University, Nanjing 210095, China

**Keywords:** *Anthurium andraeanum*, miR158, heterologous expression, *Arabidopsis*, cold stress tolerance

## Abstract

*Anthurium andraeanum* is a tropical flower with high ornamental and economic value. Cold stress is one of the major abiotic stresses affecting the quality and value of *A. andraeanum*; thus, improving the cold tolerance of this species is an important breeding objective. MicroRNAs (miRNAs) have a critical role in plant abiotic stress responses, but their specific molecular regulatory mechanisms are largely unknown, including those related to the cold stress response in *A. andraeanum*. Here, we identified and cloned the precursor of miR158 from *A. andraeanum* (Aa-miR158). Both Aa-miR158 and its target gene (*c48247*) had higher expression levels in strong leaves than in other tissues or organs. Further study revealed that the transcript level of Aa-miR158 was increased by cold stress. Heterologous overexpression of Aa-miR158 improved cold stress tolerance in *Arabidopsis*, which was associated with decreases in the malondialdehyde (MDA) concentration and relative electrical conductivity (REC) as well as increases in peroxidase (POD) and catalase (CAT) activity. Moreover, overexpressing Aa-miR158 significantly increased the expression of endogenous genes related to cold stress tolerance and reactive oxygen species (ROS) levels in transgenic *Arabidopsis* under cold stress. Overall, our results demonstrate that Aa-miR158 is significantly involved in the cold stress response and provide a new strategy for cold tolerance breeding of *A. andraeanum*.

## 1. Introduction

*Anthurium andraeanum* Lind., an important ornamental flower, originated in the tropical rainforest of South American and has been widely cultivated throughout the world. It needs to be cultivated in a high-temperature, high-humidity environment throughout the year. The optimal growth temperature of *A. andraeanum* is 20–28 °C, and cold damage symptoms occur when the temperature is below 15 °C [1]. During greenhouse cultivation of *A. andraeanum*, especially in winter, the heating costs are exorbitant; thus, improving the cold tolerance of *A. andraeanum* would help to further extend its planting range and increase its economic benefits.

MicroRNAs (miRNAs) are a class of endogenous non-coding small RNAs that negatively modulate target gene expression at the post-transcriptional level through mRNA cleavage or translational repression [2,3,4]. In plants, miRNAs regulate a broad group of transcription factors (TFs) and are involved in multiple biological processes, including development [5,6,7], biotic stresses [8] and abiotic stresses [9,10,11]. Specifically, miRNAs play a role in many horticultural traits (e.g., flowering, fruit quality, abiotic and biotic stress), and provide valuable information for future horticultural crop research and breeding [12].

Cold stress is among the major abiotic stresses and has a detrimental impact on almost every aspect of the physiology and biochemistry of plants [13]. Quite recently, many miRNAs such as miR319, miR394, miR396, miR397, miR402 and miR408 have been found to play various roles in mediating plant responses to cold stress [13,14]. The cold stress response mechanisms of miRNAs in plants involve three main pathways: (1) the C-repeat binding factor (CBF)-dependent pathway, (2) control of reactive oxygen species (ROS) accumulation, and (3) hormone signal regulation [14,15]. Specifically, miR394 and miR397 showed positive regulation of cold tolerance in plants via the CBF-dependent pathway [16,17]; miR408 has been demonstrated to enhance cold tolerance in plants through reduction of ROS levels [18]; miR165/166 conferred cold resistance by controlling ABA homeostasis [19]; and some miRNAs (e.g., miR319 and miR396) are involved in several of these pathways [20,21,22].

miR158 was one of the miRNAs firstly discovered in *Arabidopsis* [23]. Up to now, studies of miR158 have mainly concentrated on brassicaceous plant species. For example, overexpression of miR158 causes pollen abortion through the degradation of pollen contents in *Brassica campestris* [24]. In *Brassica napus*, miR158 affects cadmium (Cd) tolerance by regulating the expression of *BnRH24* under Cd exposure [25]. However, compared to other miRNAs in various species involved in abiotic stress tolerance, especially cold stress tolerance, little is known about the function of miR158 in plants.

Recently, fine-tuning miRNAs based on the regulation of target genes transcription has become a powerful biotechnological strategy to improve tolerance to biotic or abiotic stresses through the use of genetic engineering tools in plants [26]. In our previous study, miR158 and its putative target gene (designated *c48247* in our in-house collection of RNA-Seq sequences of *A. andraeanum*) were identified in *A. andraeanum* by the analysis of small RNAs [27]. In this study, the precursor of Aa-miR158 was cloned, and the sequence alignment of miR158 homologs showed that the sequence of Aa-miR158 diverged from those found in Brassicaceae. Aa-miR158 is anchored at the 3′-UTR of putative target gene *c48247*, and their sequences are completely complementary. The 5′-RLM-RACE results showed that *c48247* was not cleaved by Aa-miR158. The expression patterns of Aa-miR158 and *c48247* in different tissues of *A. andraeanum* were characterized using qRT-PCR. The expression of *Aa-miR158* was induced in *A. andraeanum* under cold stress treatment, and the heterologous overexpression of *pre-Aa-miR158* improved cold tolerance in *Arabidopsis*, indicating that Aa-miR158 is involved in the plant’s response to cold stress. To our knowledge, this is the first report demonstrating the involvement of Aa-miR158 in the cold stress response in *A. andraeanum*.

## 2. Results

### 2.1. Sequence Characterization of Aa-miR158 and Its Target Gene

**Figure 1:**
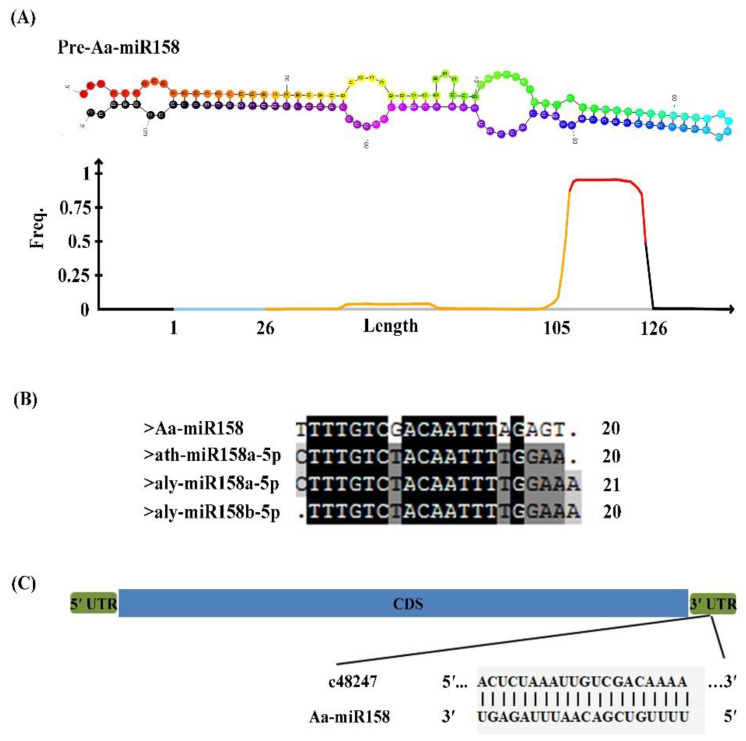
Identification of miR158 from *A. andraeanum* through small RNA sequencing. (**A**) Predicted stem–loop structure of Aa-miR158. The graph below the diagram shows the distribution of reads on the alignment to the Aa-MIR158 precursor. The red line indicates the mature sequence of Aa-miR158. (**B**) Alignment of miR158 sequences from *A. andraeanum* (Aa), *Arabidopsis lyrata* (aly), *Arabidopsis thaliana* (ath), *Brassica napus* (Bn) and *Brassica rapa* ssp. *Pekinensis* (bra). (**C**) Binding site of Aa-miR158 with its target gene *c48247*. The blue rectangle shows the *c48247* coding sequence and green rectangles show the 5′- and 3′-UTR, respectively.

Aa-miR158 was first identified from *A. andraeanum* through previous small RNA sequencing [27]. The Aa-MIR158 precursor is 164 nt long and contains a 20-nt mature sequence (5′-UUUUGUCGACAAUUUAGAGU-3′) (Figure 1). The predicted stem–loop structure based on the Aa-MIR158 precursor sequence is shown in Figure 1A. To examine the conservation of the homology of miR158, we performed multiple sequence alignment among diverse species. The result showed that, with the exception of the mismatch of six bases, Aa-miR158 has 14 conservative nucleotides that align to other species. The mature sequence of Aa-miR158 was not conserved compared with those of miR158 homologs in *Arabidopsis lyrata* (aly), *Arabidopsis thaliana* (ath), *Brassica napus* (Bn) and *Brassica rapa* ssp. *pekinensis* (bra) (Figure 1B), suggesting that Aa-miR158 might have diverse roles in *A. andraeanum* compared with those in brassicaceous plants. A previous study reported that *c48247.graph_c0* (*c48247*), a terpene synthase/tricyclene synthase gene, was the target gene of Aa-miR158 [27]. Here, we found that the mature sequence of Aa-miR158 paired with the 3′ untranslated region (UTR) of target gene *c48247* via sequence alignment (20 out of 20 bases paired) (Figure 1C). However, the expected band was not detected by the nested PCR for 5′-RLM-RACE using a 2% agarose gel electrophoresis.

### 2.2. Expression Analyses of Aa-miR158 and c48247

**Figure 2:**
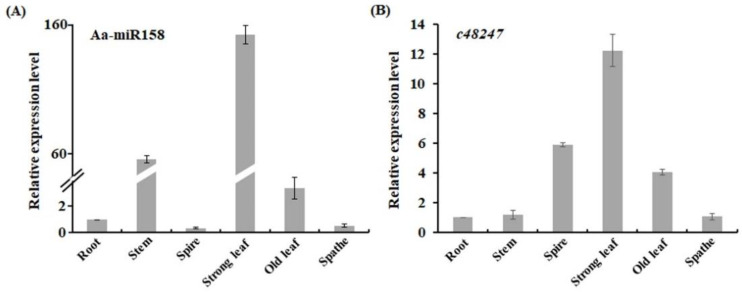
Expression patterns of Aa-miR158 (**A**) and *c48247* (**B**) in different organs of *A. andraeanum*. Error bars represent standard deviations (*n* = 3).

To explore the expression pattern of Aa-miR158, the transcript profile of mature Aa-miR158 was detected in the root, stem, spire, strong leaves, old leaves and spathe of *A. andraeanum* through qRT-PCR. Relative to the expression in the root, the expression of Aa-miR158 was highest in strong leaves (152.2-fold), followed by the stem (56.5-fold) and old leaves (3.4-fold) (Figure 2A).

Meanwhile, the transcript profile of target gene *c48247* was also detected in *A. andraeanum* using qRT-PCR. The expression level of *c48247* was highest in the strong leaves followed by the spire and old leaves (Figure 2B). The transcript level of *c48247* in the spire was nearly 6-fold that in the root, whereas Aa-miR158 had lower expression in the spire than in the root (Figure 2B). *c48247* maintained a low transcript level in the stem, which was similar to that in the root. Thus, Aa-miR158 and its target gene *c48247* were both highly expressed in the strong leaves and old leaves of *A. andraeanum*, while their expression patterns were different in the stem and spire. These results suggested that Aa-miR158 may have diverse roles in various biological processes via upregulated or downregulated expression of *c48247*.

### 2.3. Aa-miR158 and c48247 Response to Cold Stress in A. andraeanum

**Figure 3:**
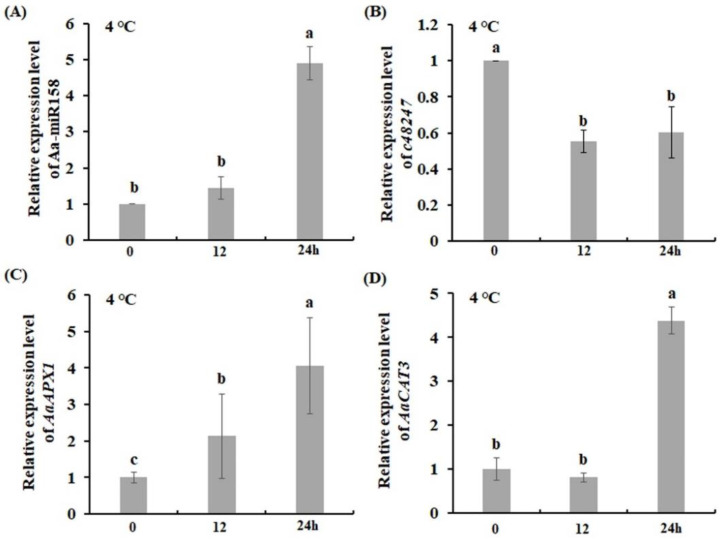
Expression analysis of Aa-miR158 (**A**), *c48247* (**B**), *AaAPX1* (**C**) and *AaCAT3* (**D**) under cold stress. Seedlings of *A. andraeanum* were cultivated in MS medium and exposed to 4 °C treatment for 0, 12 or 24 h. Error bars represent standard deviations (*n* = 3). Within each graph, values marked with the different letter are significantly different (*p* < 0.05, Student’s *t*-test).

To identify whether Aa-miR158 responds to cold stress, Aa-miR158 expression was measured in leaves of *A. andraeanum* seedlings exposed to cold stress treatment (4 °C). During 24 h of cold stress, the expression level of Aa-miR158 gradually increased and was about 5-fold higher at 24 h than at 0 h (Figure 3A). This result indicated that Aa-miR158 was involved in the cold stress response at the transcriptional level in *A. andraeanum*. On the other hand, the expression of its target gene *c48247* was downregulated at 12 and 24 h of 4 °C treatment (Figure 3B).

In addition, the expression of two cold-related genes, *ascorbate peroxidase 1* (*AaAPX1*) and *catalase 3* (*AaCAT3*), was also detected in leaves of *A. andraeanum* seedlings exposed to cold stress treatment (4 °C). The results showed that these two genes’ transcripts were significantly upregulated under cold stress treatment after 24 h, which is similar to the expression pattern of Aa-miR158 under the same cold stress conditions (Figure 3C,D).

### 2.4. Aa-miR158 Represses the Expression of c48247 in A. andraeanum

**Figure 4:**
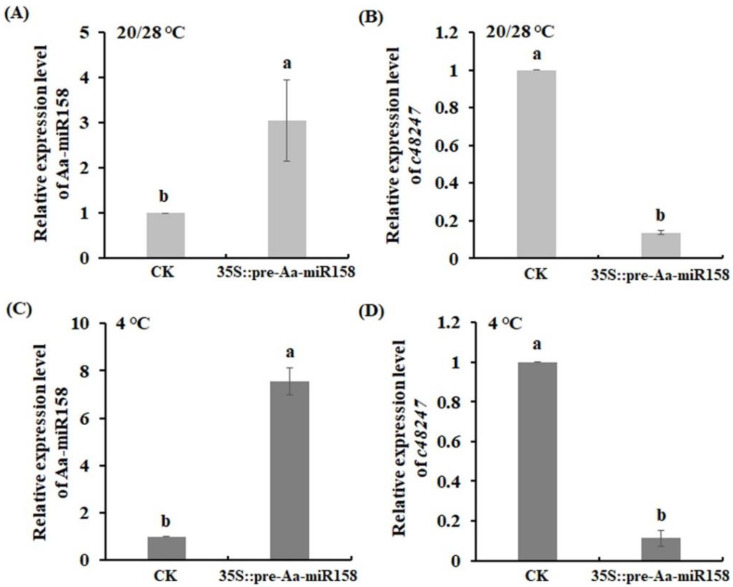
Transient overexpression assay of *pre-Aa-miR158* in *A. andraeanum* leaves. (**A**) Aa-miR158 expression at 20/28 °C; (**B**) *c48247* expression at 20/28 °C; (**C**) Aa-miR158 expression at 4 °C; (**D**) *c48247* expression at 4 °C. Error bars represent standard deviations (*n* = 3). Within each graph, values marked with the different letter are significantly different (*p* < 0.05, Student’s *t*-test).

To further investigate whether Aa-miR158 inhibited the expression of the *c48247* gene in *A. andraeanum*, a *pre-Aa-miR158* overexpression construct was infiltrated into *A. andraeanum* leaves for a transient expression assay. The transcript level of Aa- miR158 in *p35S::pre-Aa-miR158* leaves was 3-fold than the level in control leaves at 28/20 °C (Figure 4A). As expected, the expression of *c48247* was decreased in *p35S::pre-Aa-miR158* leaves compared with control leaves (Figure 4B). Interestingly, Aa-miR158 in *p35S::pre-Aa-miR158* leaves displayed a significant increase in expression of 7.6-fold at 4 °C, while *c48247* showed a significant reduction in expression of nearly 90% (Figure 4C,D). On the other hand, a co-expression assay showed that the fluorescence signal became weaker in co-expression with *pre-Aa-miR158* and a GFP reporter bearing Aa-miR158 target site compared with that in a GFP reporter bearing Aa-miR158 target site alone (Supplementary Materials, Figure S1).

### 2.5. Heterologous Expression of Aa-miR158 in Arabidopsis Improves Tolerance to Cold Stress

To investigate the biological role of Aa-miR158 in detail under different abiotic stresses, the nucleotide sequence of *pre-Aa-miR158* was amplified from genomic DNA of *A. andraeanum*. The *p35S::pre-Aa-miR158* vector was constructed and transformed into *Arabidopsis* through *Agrobacterium*-mediated transformation. A total of 11 independent T1 transgenic lines of *p35S::pre-Aa-miR158* were identified through screening on hygromycin and then confirmed via PCR. Among the 11 transgenic lines identified by hygromycin screening, the electrophoresis band corresponding to Aa-miR158 was detected in all 11 lines but not in WT plants (Supplementary Materials, Figure S2A). Aa-miR158 was found to be overexpressed at high levels in two transgenic lines (designated as *p35S::pre-Aa-miR158*-*1* and -*5*), which were used for further analysis (Supplementary Materials, Figure S2B).

Considering that miRNAs are involved in various stress responses [28,29], we investigated the responses of two *p35S::pre-Aa-miR158* overexpressing transgenic *Arabidopsis* lines to salinity, drought and cold stress. Compared with WT, the phenotypes of *p35S::pre-Aa-miR158* transgenic plants displayed no marked changes under salt stress (Supplementary Materials, Figure S3A). As expected, the MDA levels and REC exhibited no significant differences between WT and *p35S::pre-Aa-miR158* transgenic lines under salt stress (Supplementary Materials, Figure S3B). We next tested the function of Aa-miR158 under drought stress in transgenic and WT plants. The soil moisture content decreased by nearly 90% in the drought treatment compared with that in the control (Supplementary Materials, Figure S4B). There was also no clear effect of Aa-miR158 overexpression on the phenotype of *p35S::pre-Aa-miR158* transgenic plants after withholding water (Supplementary Materials, Figure S4A). Under drought stress, MDA levels were significantly lower in the two *p35S::pre-Aa-miR158* transgenic lines than in WT, but the soluble protein content showed no significant difference, and the POD and CAT activities were even lower in one or both of the *p35S::pre-Aa-miR158* transgenic lines than in WT plants (Supplementary Materials, Figure S4C).

**Figure 5:**
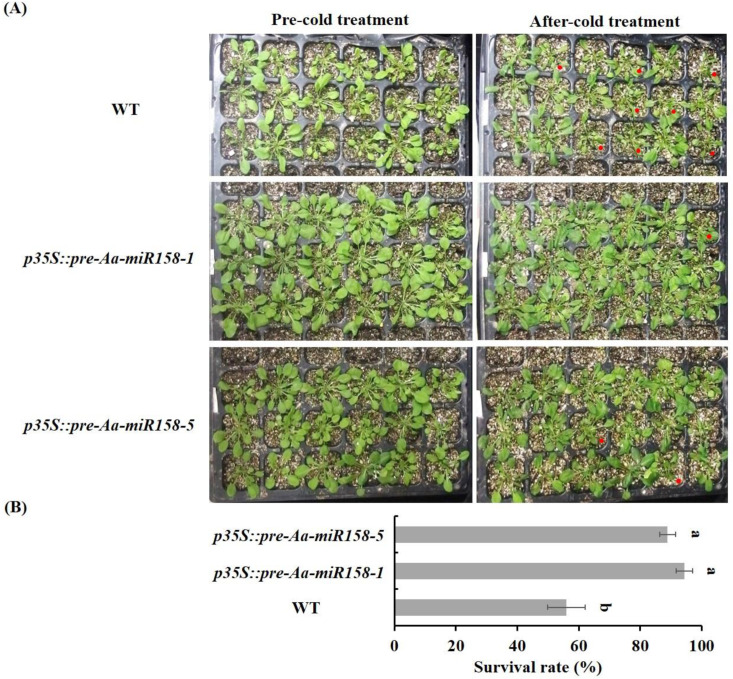
Comparative analyses of Aa-miR158 transgenic (*p35S::pre-Aa-miR158*-1 and *p35S::pre-Aa-miR158*-5) and wild-type (WT) *Arabidopsis* plants under cold stress. (**A**) Phenotype of Aa-miR158 transgenic and WT *Arabidopsis* plants. Red dots indicate severely damaged plants. (**B**) Survival rates after cold stress treatment for 24 h. Error bars represent standard deviations (*n* = 3). Values marked with the different letter are significantly different (*p* < 0.05, Student’s *t*-test).

To assess the cold stress tolerance of transgenic *Arabidopsis*, we investigated the phenotypic characteristics of *p35S::pre-Aa-miR158* and WT plants under cold stress (4 °C for 6 h followed by 0 °C for 1 h) and normal growth conditions (22/20 °C, 12/12 h). Under normal conditions, no marked phenotypic differences were found between transgenic lines and WT plants (Figure 5A). Under cold stress, the leaves of WT *Arabidopsis* began to turn yellow and showed severe wilting, which eventually led to their death. However, *p35S::pre-Aa-miR158* transgenic plants exhibited enhanced cold tolerance and a low degree of chilling injury compared with WT plants (Figure 5A). Following the exposure to cold stress treatment, the survival rate of two *p35S::pre-Aa-miR158* transgenic lines was >80%, which was significantly higher than that of WT (<60%) (Figure 5B).

**Figure 6:**
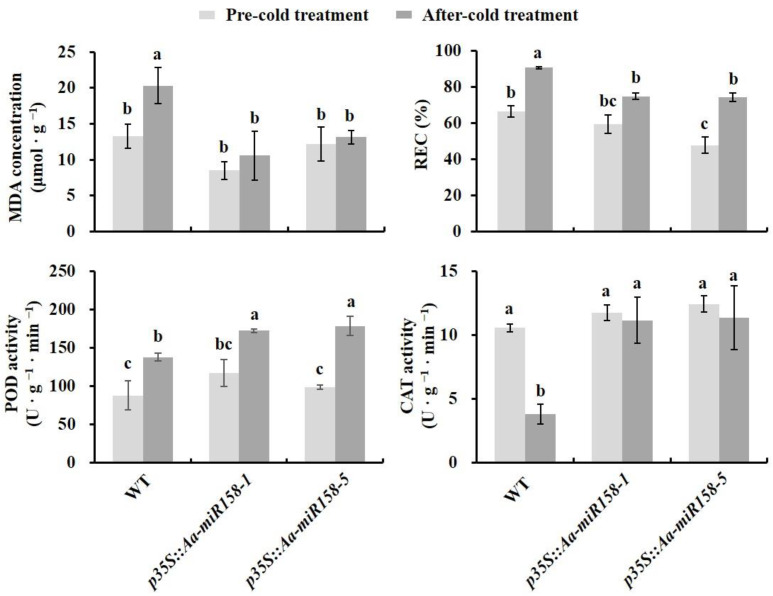
Statistical analyses of malondialdehyde (MDA) concentration, relative electrical conductivity (REC), and activity of peroxidase (POD) and catalase (CAT) in leaves of *p35S::pre-Aa-miR158* transgenic and WT lines under cold stress. Error bars represent standard deviations (*n* = 3). Within each graph, values marked with the different letter are significantly different (*p* < 0.05, Student’s *t*-test).

The levels of MDA were significantly lower under cold stress in *p35S::pre-Aa-miR158* plants than in WT plants (Figure 6). REC was significantly lower under cold stress in *p35S::pre-Aa-miR158* plants than in WT, whereas POD and CAT activities were significantly higher (Figure 6). These results suggested that *p35S::pre-Aa-miR158* transgenic lines are more resistant to cold stress than WT plants.

### 2.6. Aa-miR158 Improves Cold Stress Tolerance by Affecting Expression of Genes Related to Cold Stress and ROS

**Figure 7:**
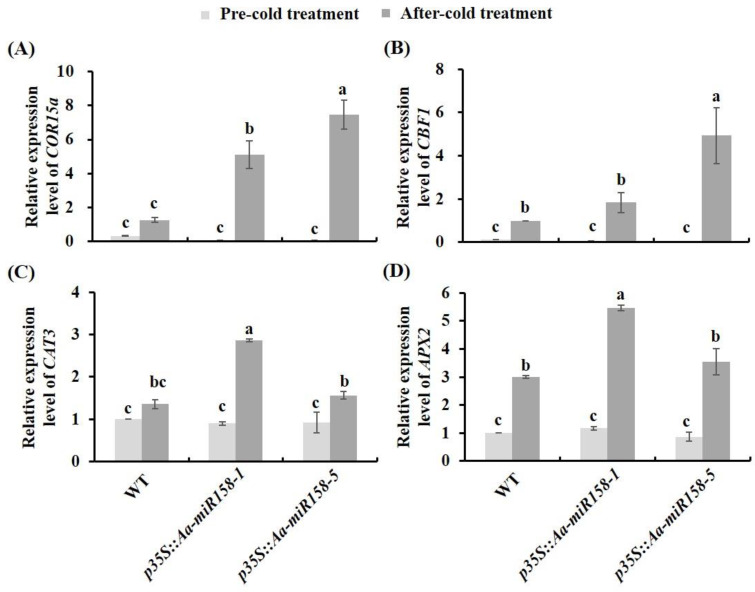
Relative expression levels of genes related to cold stress and ROS in leaves of *pre-Aa-miR158* transgenic (*p35S::pre-Aa-miR158*-1 and *p35S::pre-Aa-miR158*-5) and wild-type (WT) *Arabidopsis* plants under cold stress. (**A**): *COR15a*; (**B**): *CBF1*; (**C**): *CAT3*; (**D**): *APX2*. Error bars represent standard deviations (*n* = 3). Within each graph, values marked with the different letter are significantly different (*p* < 0.05, Student’s *t*-test).

In *Arabidopsis*, both *cold-regulated 15a* (*COR15a*) and *C-REPEAT/DRE BINDING FACTOR 1* (*CBF1*) are involved in chilling tolerance [30,31]. In this study, the expression of *COR15a* and *CBF1* was not significantly different between WT and *p35S::pre-Aa-miR158* transgenic *Arabidopsis* under unstressed (control) conditions (Figure 7A,B). Nevertheless, increases in transcript level in transgenic *p35S::pre-Aa-miR158* compared to WT plants were observed for both *COR15a* and *CBF1* under conditions of cold stress (Figure 7A,B). In *p35S::pre-Aa-miR158-1*, the expression level of *COR15a* under cold stress was almost 5.0-fold that in WT, and in *p35S::pre-Aa-miR158-5*, the expression levels of *COR15a* and *CBF1* under cold stress were 7.5-fold and 4.9-fold those in WT, respectively (Figure 7A,B). In addition, the expression levels of *CAT3* and *APX2* in two *p35S::pre-Aa-miR158* transgenic lines were not significantly different from those in WT under unstressed (control) conditions, while under cold stress there was a significant increase in the transcript levels of both genes in *p35S::pre-Aa-miR158-1* relative to WT (Figure 7C,D). These results illustrated that Aa-miR158 may improve cold stress tolerance by activating genes related to cold stress and ROS at the transcriptional level, emphasizing the important function of Aa-miR158 in plant survival.

## 3. Discussion

miRNAs are involved in many kinds of abiotic stresses in various species; however, there are very few studies on the function of miRNAs in *A. andraeanum*. In this study we isolated Aa-miR158, a homolog of the miR158 family, which was previously reported to be a specific miRNA in Brassicaceae. The mature sequence of Aa-miR158 was poorly aligned (<70%) with miR158 sequences in other species and its highest expression was found in strong leaves, suggesting that the function of Aa-miR158 may have diverged during evolution (Figure 1 and Figure 2). Actually, more than one member of the miR158 family exist in other plant species [32], while how many members are included in the miR158 family in *A. andraeanum* and their expression patterns in *A. andraeanum* remain unclear and need to be investigated in the future.

Low-temperature stress damages plant tissue and thus affects the growth and development of important ornamental flowers [33]. Our expression analysis showed that the transcript level of Aa-miR158 and cold-related genes was increased by low temperature (Figure 3), suggesting Aa-miR158 may be involved in the cold stress signaling pathway and a possible correlation between elevated levels of Aa-miR158 and enhanced cold tolerance in *A. andraeanum*.

Aa-miR158 was predicted to be an unconservative miRNA and a terpene synthase (TPS) gene, *c48247*, was predicted to be the target of Aa-miR158 [27]. The fluorescence assays showed that Aa-miR158 can silence the GFP reporter bearing Aa-miR158 target site (Supplementary Materials, Figure S1), while the 5′-RLM-RACE results showed that the target site was not cleaved by Aa-miR158, providing evidence that Aa-miR158 might regulate the function of *c48247* by repressing its translation in *A. andraeanum*. However, each miRNA corresponds to more than one target genes in general; therefore, whether Aa-miR158 has other potential target genes, especially related to cold stress tolerance, also needs to be further explored.

The CBF-dependent pathway has been proved to play a leading role in plant tolerance to low-temperature stress [34]. Overexpression of *pre-Aa-miR158* in *Arabidopsis* enhanced cold stress tolerance compared with that of WT plants (Figure 5). In addition, the expression levels of endogenous cold stress-related genes (*COR15a* and *CBF1*) were significantly higher in *p35S::pre-Aa-miR158* transgenic plants than in WT under cold stress, except for *CBF1* in *p35S::pre-Aa-miR158-1* (Figure 7). Our results suggested that Aa-miR158 enhanced low-temperature tolerance in *Arabidopsis* by activating the expression of genes involved in the CBF-dependent pathway.

The production and removal of ROS maintains a balance in plants under normal physiological conditions [35,36]. However, cold stress leads to the excess accumulation of ROS, and this damages intracellular components and membrane lipids [37]. Our results showed that MDA levels and REC were significantly lower in *p35S::pre-Aa-miR158* transgenic plants than in WT under cold stress (Figure 6). They also showed that the POD and CAT activity were significantly higher in *p35S::pre-Aa-miR158* transgenic plants than in WT under cold stress (Figure 6). In addition, the transcript levels of *CAT3* and *APX2* were significantly higher in *p35S::pre-Aa-miR158-1* than in WT under cold stress (Figure 7). These results suggested that Aa-miR158 improves low-temperature tolerance in plants, possibly due in part to the reduction of ROS damage.

Interestingly, although *p35S::pre-Aa-miR158* transgenic plants did not appear to have significantly improved the tolerance to other abiotic stresses compared with WT, the MDA content decreased significantly compared with WT under drought stress (Supplementary Materials, Figures S3 and S4). The above results imply that the increased tolerance caused by the overexpression of *pre-Aa-miR158* may be highly specific to cold stress in *A. andraeanum*, but its possible effects on other stress response processes need to be considered in future investigations.

Although *Arabidopsis* transgenics that overexpressed *pre-Aa-miR158* showed more tolerance to cold treatment in this study, many of the details of the regulatory mechanisms of Aa-miR158 remain elusive. A recent review showed that miRNAs respond to low-temperature stress by directly or indirectly responding to external stimuli by mediating target genes, or responding to multiple stresses, and thus starting the hydrolysis or redox processes [38]. In various species, the regulatory mechanisms of the miRNAs involved in low-temperature stress were different, for example, phytohormone signal transduction and the ROS scavenging pathway [20,39,40]. Therefore, the specific regulation mechanism of Aa-miR158 involved in cold tolerance in heterologous *Arabidopsis* requires further explication by experimental evidence such as degradome sequencing.

With the development of omics technology, the miRNAs sequence information can be obtained more conveniently by high-throughput sequencing. However, the stable genetic transformation of *A. andraeanum* remains difficult, is very time-consuming and has quite a low transformation efficiency. It is limited for investigating the potential function of miRNAs in *A. andraeanum*. In future research, the functional characterization of Aa-miR158 will be further investigated in *A. andraeanum* by establishing a rapid and efficient genetic transformation system, which will lay the foundation for utilizing miRNAs to improve stress resistance in other ornamental plants.

## 4. Materials and Methods

### 4.1. Plant Materials and Growth Conditions

In vitro plantlets of *A. andraeanum* cultivar ‘Sonate’ were grown in a tissue culture room under 12/12 h, 25/25 °C, day/night conditions at Nanjing Agricultural University. The potted plants were grown in the Baima Teaching and Research Base of Nanjing at 25/20 °C (day/night), 70–80% relative humidity, and natural lighting under standard greenhouse conditions. Leaves from plant tissue culture and different organs (root, stem, spire, strong leaves, old leaves and spathe) from potted plants were collected from at least three independent plants for further analysis. After collection, the samples were frozen immediately in liquid nitrogen and stored at −80 °C until use.

The *A. thaliana* Columbia (Col-0) ecotype was used for transgenic analysis. The seeds were surface-sterilized in 75% alcohol with 0.03% Triton X-100 for 2 min, rinsed three times in 70% alcohol and then washed three times in distilled water. Disinfected seeds were sown on Murashige and Skoog (MS) medium with 1% sucrose and placed in a 4 °C refrigerator for 3 days. After refrigeration, plates with seeds were transferred to a light incubator under a 12 h photoperiod with day/night conditions at 22/20 °C. The seedlings were transplanted into soil at the two-leaf stage.

### 4.2. Sequence Analysis

The precursor and mature sequences of Aa-miR158 were acquired from our in-house small RNA sequencing library (Bioproject, PRJNA472856). The corresponding target gene was predicted using the plant small RNA target (psRNA Target) online sever (http://plantgrn.noble.org/psRNATarget/, accessed on 20 September 2016; now available at https://www.zhaolab.org/psRNATarget/, accessed on 25 May 2018) and verified by small RNA-mRNA co-analysis. Genomic DNA was isolated from leaves using a cetyltrimethylammonium ammonium bromide (CTAB) method for the amplification of the *pre-Aa-miR158* sequence. The *pre-Aa-miR158* fragment was amplified by PCR using KOD-Plus-Neo (TOYOBO, Osaka, Japan). The PCR reaction conditions were as follows: initial denaturation at 94 °C for 3 min; 36 cycles of denaturation at 98 °C for 10 s, annealing at 55 °C for 30 s, and extension at 72 °C for 10 s; and final extension at 72 °C for 7 min. Multiple sequence alignment was performed with DNAMAN software (Lynnon Biosoft, CA, USA). The primers used for *pre-Aa-miR158* cloning are listed in the Supplementary Materials, Table S1.

### 4.3. Expression Analysis

Total RNA was extracted using an improved CTAB method [41] and then reverse-transcribed with a Mir-X miRNA First-Strand Synthesis Kit (Takara, Kyoto, Japan) using oligo (dT) primers. Aa-miR158–specific forward primers and a universal reverse primer (mRQ3′ primer) supplied by the manufacturer were used. Primers for target genes were designed by Primer Premier 5.0 and synthesized by Tsingke Biotechnology Company (Nanjing, China). SYBR^®^ Premix Ex Taq™ (Takara, Japan) was used for qRT-PCR. The 5S ribosomal RNA and a housekeeping gene, glyceraldehyde-3-phosphate dehydrogenase (*Aa-GAPDH*), were used as internal references for the quantification of miRNA and target gene expression, respectively [42,43]. qRT-PCR was performed using a QuantStudio™ 3 Real-Time PCR System (ABI, MA, USA) according to the manufacturer’s instructions. The relative expression level was calculated using the 2^−ΔΔCt^ method [44] and the standard deviation was calculated using three biological replicates. The *t*-test (*p* < 0.05) was selected for statistical analysis. The primers used for qRT-PCR are listed in the Supplementary Materials, Table S1.

Leaves from wild-type (WT) and *p35S::pre-Aa-miR158* transgenic *Arabidopsis* were frozen in liquid nitrogen and stored at −80 °C for qRT-PCR analysis. Total RNA extraction was performed as described above, and cDNA was synthesized using a PrimeScript RT reagent Kit with gDNA Eraser (Takara, Japan). *Arabidopsis tubulin2* (*AtTUB2*) was used as the qRT-PCR internal control. The primers used for qRT-PCR are listed in the Supplementary Materials, Table S1.

### 4.4. Transient Overexpression of Pre-Aa-miR158 via Agrobacterium Injection

The pGreenII 62-SK vector (853 bp) was used for the transient overexpression of *pre-Aa-miR158* in leaves of *A. andraeanum*. The recombinant overexpression vector *p35S::pre-Aa-miR158* was transformed into *Agrobacterium* strain ‘GV3101 (psoup)’ and then infiltrated into the leaves. The *Agrobacterium*-infiltrated plants were incubated in a dark chamber at room temperature for 1 d and then in a natural greenhouse at 28/20 °C (day/night) or a temperature chamber at 4 °C, 12h/12h for 2 d. The samples were collected and frozen immediately in liquid nitrogen for RNA extraction and qRT-PCR analysis. Three biological replicates with three independent plants per replicate were included for the expression analysis of each gene. The primers used for transient overexpression are listed in the Supplementary Materials, Table S1.

### 4.5. Fluorescence Assay

The green fluorescent protein (GFP)-fused the 3′UTR of target gene was inserted into the plasmid pGREEN through the *Xba*I and *Sma*I cleavage sites. The fused protein p35S::c48247-GFP plasmid was transformed into *Agrobacterium* tumefaciens ‘GV3101 (psoup)’ and then individually transfected and co-transfected with *p35S::pre-Aa-miR158* plasmid into tobacco leaves (OD600 = 1.0), respectively. A fluorescence microscope (Leica DM6, Weztlar, Germany) was used to take images at 460–500 nm wavelengths for GFP. The primers for the fluorescence assay are listed in the Supplementary Materials, Table S1.

### 4.6. 5′-RLM-RACE Assay

5′-RLM-RACE was performed to determine the cleavage site of target using the FirstChoice RLM-RACE Kit (Ambion, TX, USA). The total RNA that removed genome DNA was ligated with a 5′-adaptor and reversed using random primers to obtain 5′-RACE cDNA. Nested PCR was performed using adaptor-derived and gene-specific primers to obtain the 3′-cleavage products. The primers used for the 5′-RLM-RACE PCR are listed in the Supplementary Materials, Table S1.

### 4.7. Vector Construction and Plant Transformation

The full-length of *pre-Aa-miR158* was cloned into the pCAMBIA1302 vector behind the 35S promoter by using the *Bgl*II and *Spe*I cleavage sites. The construct was then introduced into *Agrobacterium* strain EHA105 and transformed into *Arabidopsis* WT (Col-0) plants through the floral-dip method [45]. The transgenic plants were screened on MS medium with 35 mg·L^−1^ hygromycin and confirmed by PCR amplification assay to detect the *p35S::pre-Aa-miR158* transgene lines. The acquired hygromycin-resistant plants were used for further analysis. The primers used for producing the overexpression vector and DNA detection are listed in the Supplementary Materials, Table S1.

### 4.8. Abiotic Stress Treatments

In vitro plantlets of *A. andraeanum* cultivar ‘Sonate’ were used to examine the effects of cold stress on Aa-miR158 and *c48247* gene expression. The plantlets were placed in a temperature chamber at 4 °C for 0, 12 or 24 h. After treatment, 0.1–0.2 g of leaves was harvested and immediately frozen in liquid nitrogen for expression analysis. The cold stress treatment assay was performed three times.

To examine the effects of *pre-Aa-miR158* overexpression on stress tolerance, the surface-sterilized seeds of *Arabidopsis* WT plants and *p35S::pre-Aa-miR158* transgenic plants were sown on MS medium (1% sucrose). For cold stress treatment, 3-week-old transgenic and WT plants grown in soil were placed in a light incubator at 4 °C for 6 h followed by 0 °C for 1 h. Phenotypic observation, physiological index determination and gene expression analysis were performed after the cold stress treatment. The survival rate was determined after resuming growth under a 12 h photoperiod and 22/20 °C light/dark temperatures for 24 h. For the salt stress treatment, 3-week-old transgenic and WT plants grown in Hoagland’s nutrient solution with 150 mM NaCl were placed in a light incubator (22/20 °C, light/dark and 12 h photoperiod) for 3 d. For drought stress treatment, 3-week-old transgenic and WT plants grown in a light incubator (22/20 °C light/dark and 12-h photoperiod) were subjected to natural drought (withholding water) treatment for 21 d.

### 4.9. Determination of Physiological Indexes

The malondialdehyde (MDA) content was determined by the thiobarbituric acid (TBA) method [46] and the peroxidase (POD) activity and catalase (CAT) activity were determined by the guaiacol chromogenic method and ultraviolet absorption method, respectively [46]. The content of soluble protein was determined by the Coomassie brilliant blue G-250 staining method [46]. The soil moisture content was determined by the oven-drying method. The relative electrical conductivity (REC) was determined with a digital conductivity meter (WTW, Munich, Germany). All measurements were performed on three biological replicates and significance analyses were performed using SPSS 10.0 software (IBM, NY, USA).

## 5. Conclusions

In this study, we isolated and cloned the nucleotide sequence of *pre-Aa-miR158* from *A. andraeanum* and found that Aa-miR158 was involved in cold stress tolerance by heterologous overexpressing assay in *Arabidopsis*. To our knowledge, this is the first study to identify the function of Aa-miR158 in abiotic stress tolerance. The findings of this study provide a theoretical guide for greenhouse production and breeding work through the fine-tuning of microRNAs on their target genes transcription to improve abiotic stress tolerance in anthurium.

## Supplementary Materials

The following supporting information can be downloaded at: https://www.mdpi.com/article/10.3390/plants11233371/s1. Figure S1: Fluorescence assay. Co-expression with *pre-Aa-miR158* and a GFP reporter bearing Aa-miR158 target site. Bars = 100 μm. Figure S2: DNA and RNA identification of *pre-Aa-miR158* transgenic *Arabidopsis*. (A) DNA identification. M, marker; H_2_O, negative control; P-, empty vector; P+, positive control; WT, wild-type; #1–#11, Aa-miR158 transgenic lines. (B) Transcript levels of Aa-miR158 in transgenic lines #1 and #5. Error bars represent standard deviations (*n* = 3). Figure S3: Comparative analyses of Aa-miR158 transgenic (*p35S::pre-Aa-miR158*-1 and *p35S::pre-Aa-miR158*-5) and wild-type (WT) *Arabidopsis* plants under salinity stress. (A) Phenotypes of Aa-miR158 transgenic and WT *Arabidopsis* plants. (B) Malondialdehyde (MDA) concentration and relative electrical conductivity (REC) in Aa-miR158 transgenic and WT lines. Error bars represent standard deviations (*n* = 3). Within each graph, values marked with the different letter are significantly different (*p* < 0.05, Student’s *t*-test). Figure S4: Comparative analyses of Aa-miR158 transgenic (*p35S::pre-Aa-miR158*-1 and *p35S::pre-Aa-miR158*-5) and wild-type (WT) *Arabidopsis* plants under drought stress (water withheld for 21 d). (A) Phenotype of Aa-miR158 transgenic and WT *Arabidopsis* plants. (B) Soil moisture content. (C) Malondialdehyde (MDA) concentration, soluble protein (SP) content, peroxidase (POD) activity and catalase (CAT) activity in Aa-miR158 transgenic and WT lines. Error bars represent standard deviations (*n* = 3). Within each graph, values marked with the different letter are significantly different (*p* < 0.05, Student’s *t*-test). Table S1: Primers used in this study.
