# Supplementary material for: Identification of microRNA158 from Anthurium andraeanum and Its Function in Cold Stress Tolerance
Source: Plants (Basel). 2022 Dec 4;11(23):3371. doi: 10.3390/plants11233371 (PMC9735552; doi:10.3390/plants11233371)
Supplement: Supplementary file 1 [file plants-11-03371-s001.zip › plants-1994346-supplementary.pdf]

Supplementary Table S1 Primers used in this study

Primer name	Sequence (5′–3′)
**Primers for cloning**	
Pre-Aa-miR158-F	GAAGTTTTTGTCAACAATTCCTTGTTAGT
Pre-Aa-miR158-R	AAAAGGATATGGATTTGTCAGCGA
**Primers for producing overexpression vector**	
Pre-Aa-miR158m-F	gaagatctGAAGTTTTTGTCAACAAT
Pre-Aa-miR158m-R	actagtAAAAGGATATGGATTTGTCAGC
**Primers for transient overexpression vector**	
Pre-Aa-miR158-SK62-F	CGGTGGCGGCCGCTCTAGAACTAGTGaagtttttgtcaacaat
Pre-Aa-miR158-SK62-R	CGAATTCCTGCAGCCCGGGGGATCCaaaaggatatggatttgtcagc
**Primers for GFP vector**	
c48247Tar-pGreen-F	CTAGAATGgACTCTAAATTGTCGACAAAACCC
c48247Tar-pGreen-R	GGGTTTTGTCGACAATTTAGAGTccatT
**Primers for qRT-PCR**	
Aa-miR158q-F	CGGTTTTGTCGACAATTTAGAGTC
5SrRNA-F	GATGCGATCATACCAGCACTAA
5SrRNA-R	GATGCAACACGAGGACTTCCC
c48247-F	CTCTGGTCTCCTCAATGCCATAAGC
c48247-R	GTAGTCGCTCGTCAAGGTCTGAATG
AaAPX1-F	CTCAGCCACGGGGCCAACAA
AaAPX1-R	GGGATCTCAGGCCCTCCGG
AaCAT3-F	CGCCCTTCCAGCGCGTATGA
AaCAT3-R	ATGCGCTCCCGAGCGAAGT
AaGADPH-F	CCGAGTTCCCACTGTCGATG
AaGADPH-R	AATGCTCGACCTGCTGTCAC
AtCOR15a-F	GCAGATGGTGAGAAAGCGAAAGAC
AtCOR15a-R	CGGCTTCTTTTCCTTTCTCCTC
AtCBF1-F	GGCGTTGGCTTTTCAAGATG
AtCBF1-R	AAGTCGGCATCCCAAACATT
AtCAT3-F	AATTCAACGGCGGAGGTCAT
AtCAT3-R	AGCGTCAATGGCACTACCAA
AtAPX2-F	TCAGGATTCGAGGGTGCATG
AtAPX2-R	AAGGCATCCTCATCTGCAGC
AtTUB2-F	CCAGGGTGGTCAATGCG
AtTUB2-R	TCCCAGGCTCCAAATCC
**Primers for DNA detection**	
pCAMBIA1302-F	ATTGTCGTTTCCCGCCTTC
Aa-miR158i-R	AAAAGGATATGGATTTGTCAGCGA
**Primers for 5′-RLM-RACE PCR**	
Gene-specific outer primer-R	TCAACAATTCCTTGTTAGTCGTT

Gene-specific inner primer-R	ATATGCCAACGGAACTTGTGTC
